# Isotherm and kinetic investigations of sawdust-based biochar modified by ammonia to remove methylene blue from water

**DOI:** 10.1038/s41598-023-39971-0

**Published:** 2023-08-05

**Authors:** Mohamed A. Hassaan, Murat Yılmaz, Mohamed Helal, Mohamed A. El-Nemr, Safaa Ragab, Ahmed El Nemr

**Affiliations:** 1 National Institute of Oceanography and Fisheries (NIOF), Kayet Bey, Elanfoushy, Alexandria, Egypt; 2 Department of Chemical Engineering, Faculty of Engineering, Osmaniye Korkut Ata University, 80000 Osmaniye, Turkey; 3 Department of Chemical Engineering, Faculty of Engineering, Minia University, Minia, 61519 Egypt

**Keywords:** Environmental chemistry, Pollution remediation, Chemical engineering

## Abstract

Chemical industry effluent may pose significant environmental risks to both human health and the economy if it is not properly managed. As a result, scientists and decision-makers are paying increasing attention to developing a sustainable, low-cost wastewater treatment technique. This work aims to investigate the adsorption of Methylene Blue (MB) dye present in water using biochar derived from sawdust modified by boiling in an ammonia solution (SDBA). The properties of SDBA were characterized by BET, SEM, XRD, BJH, FT-IR, DTA, EDX and TGA analyses. The presence of –OH and –NH groups in SDBA was confirmed by FTIR, which proved that the NH_4_OH treatment of biochar successfully added nitrogen groups on its surface. The influence of pH (2 to 12), MB dye initial concentration (20 to 120 mg/L), adsorbent dosage (0.5 to 4.0 g/L) and contact time (0 to 180 min) on the adsorption process has been investigated. The adsorption of MB dye is more favorable at basic pH, with optimum adsorption at pH 8. Using a starting concentration of 20 mg/L of MB dye and a 4.0 g/L SDBA dose, the maximum percent clearance of MB dye was 99.94%. Experimental results were fitted to the Freundlich (FIM), Tempkin (TIM) and Langmuir (LIM) isotherm models (IMs). The FIM fitted the equilibrium data well, with a 643.74 mg/g* Q*_m_. Various error function models were used to test the data obtained from IMs. According to Error Function results, experimental data showed that it fits better for LIM and FIM. Kinetic studies indicated that the MB dye adsorption procedure followed pseudo-second-order (PSOM) kinetics based on film diffusion (FDM), pseudo-first-order (PFOM) and intra-particle diffusion models (IPDM). MB dye sorption on the SDBA involved electrostatic interaction, surface participation, hydrogen bond and π–π interactions. The adsorption mechanism of MB dye by SDBA was proposed as physical adsorption via the electrostatic attraction process. SDBA is an effective adsorbent in removing MB dye from water. Six adsorption–desorption cycles of the MB dye were run through the regeneration of SDBA with only a minimal amount of adsorption capacity loss, demonstrating the reusability of manufactured SDBA.

## Introduction

Industrial effluents are one of the primary sources of environmental pollution^1^. Several industries, including the textile, dyeing, food, drug, paper, leather, and cosmetics industries, release colored wastewater^1^. Among these industries, the dye industry is of great importance. Methylene blue is a kind of synthetic high-chromatic dye that is water soluble and widely used in chemical indicators, dyes, biological stains and pharmaceuticals. It is readily visible at room temperature and stable in water^2,3^. The bulk of these dyes poses substantial environmental issues due to the buildup of carcinogenic substances and the release of poisonous substances^4^. Due to dyes' resistance to biodegradation and toxic nature, much recent interest has been in their removal from wastewater^5^. Because dyes are recognized to be hazardous and persistent environmental contaminants, dye degradation must be accomplished via physical and chemical techniques^2,6^. As a result, wastewater treatment has emerged as a very busy study area. To remove contaminants from contaminated water, several techniques (such as ion exchange, membrane filtration, catalytic degradation, biological treatment, coagulation, chemical precipitation and adsorption) have been proposed and developed^7^. Adsorption is frequently employed since it is straightforward and efficient^8,9^. Activated carbon is the primary adsorbent used to treat various dyes^10,11^. However, because of its high added value and difficulty in regeneration, the usage of activated carbon is subject to various limitations^11,12^. The use of low-cost raw materials has been studied as a solution to this issue, including food waste^3^, barley straw^13^, corncob^14^, prickly pear^15^, Pitaya fruit^16^, cocoa leaves^17^, rice husk^18^, mandarin waste^19^, peach stones^20^, pinewood^21^, almond shell^22^, olive stone waste^23^. By pyrolyzing this biomass source, activated carbon can be created with either chemical activation (for example, ZnCl_2_, H_3_PO_4_) or physical (for example, H_2_O, CO_2_, O_2_), producing materials with a large surface area^24^. Biochars have more functional groups compared to activated carbons while having a smaller surface area and pore volume^25,26^. The number and type of functional groups incorporated into the biochars' structure will directly affect how effectively they remove wastewater. By modifying the surface of the biochar chemically, this can be accomplished. The adsorption capacity of biochars can be increased via various techniques, including nanoscale creation, carbon surface activation, oxidation and metal impregnation^27^. Attaching amino groups to the surface of biochar can improve its ability to absorb substances^26^. By treating biochar with various acids (H_2_SO_4_, H_3_PO_4_, or HNO_3_), bases (KOH, H_2_SO_4_, H_3_PO_4_, NaClO or HNO_3_), or oxidizing agents ((NH_4_)_2_ S_2_O_8_, KMnO_4_, H_2_O_2_, NaClO or NH_3_H_2_O), it is possible to enhance the number of functional groups^25,28,29^. Biochars are very thermally stable and are loaded with nanometals, which improve oxidation resistance, increase specific surface area, extend adsorption sites, and increase specific surface area^30^. Aniline is one of the most often used reducing agents, along with H_2_, FeSO_4_, Na_2_SO_3_, and NH_3_H_2_O^31^, but the selection of each reducing agent might vary depending on the reaction's conditions, the type of dyes utilised, and the desired reduction performance. Increased surface functional groups and improved stability will boost the biochar's adsorption capability and reusability, which is the justification for using ammonia to alter the material for MB dye removal. This approach to wastewater treatment is novel and looks promising, having possible applications in many other fields.

Numerous elements, including its high carbon content, aromatic functional groups containing oxygen, and high porosity, may impact its structure. Its surface area, stable molecular structure, and porosity encourage the adsorption of contaminants on it^32^. In the first 15 min at starting pH 2, direct red 23 and 80 were shown to have adsorption capacities of 10.72 and 21.05 mg/g for biochar made from maize stover, according to Fuertes et al.^33^. Wang et al.^34^ sought to examine the hazardous metals' capacity to bind to hickory wood-derived biochar after it had been KMnO_4_-treated. According to their findings, the biochar they created had an adsorption capacity for Pb of 153.1 mg/g, Cu of 34.2 mg/g, and Cd of 28.1 mg/g. This variation in adsorption could be brought about by these metals' varying valences and their affinity for biochar.

Additionally, Sun et al.^35^ observed that increasing the amount of biochar (made from swine dung) from 1 to 8 g/L led to more active sites that could be used to adsorb methylene blue. By increasing the quantity of biochar used for the adsorption of organic contaminants, Wang et al.^36^ discovered comparable outcomes. When using Peanut straw biochar to remove methyl violet at concentrations ranging from 40 to 816 mg/L, Xu et al.^37^ achieved an adsorption capacity of 104.61 mg/g.

Many studies deal with the removal of MB dye through adsorption. However, to the author's knowledge, the modification of sulfonated SD biochar by treatment with NH_4_OH (SDBA) is used for the first time for MB dye removal.

Thus, in this study, the effectiveness of a novel, readily available, biodegradable, non-hazardous, and reasonably priced adsorbent (sawdust biochar modified with ammonia, or SDBA) made from sawdust was investigated for the adsorption of MB dye from water. The amount of research on the adsorption of MB dye using sawdust-derived biochar in the Literature is fairly small. It was assessed how well SDBA removed MB dye from wastewater. The impact of SDBA dose, pH, starting MB dye concentration, and contact time between SDBA and MB dye were examined as elimination conditions for MB dye from water. Adsorption isotherms and kinetics were also examined in this study to ascertain the SDBA adsorbent's maximum adsorption capacity and adsorption mechanism.

## Materials and methods

### Resources and instrument

Wood sawdust from the wood market in Alexandria, Egypt, was obtained and used as a starting material to fabricate biochar. Methylene blue dye (Assay 99%) of Sigma Aldrich in the USA was used to create the standard stock solution. This investigation used a Pg Instrument model T80 UV/Visible High-Performance Double Beam Spectrophotometer, PG Instruments Limited, Woodway Lane, Alma Park, Leicestershire LE17 5BH, United Kingdom^38^. A JS Shaker (JSOS-500) JS Research Inc., South Korea and pH meter JENCO (Model 6173, USA) were used in the experimental work. The adsorption–desorption isotherm of SDB, and SDBA biochars was conducted in N_2_ environment based on a thermodynamic model. N_2_ adsorption at 77 K was used to determine the surface area (SA) of the SDB and SDBA using BELSORP-Mini II, BEL Japan, Inc.^39,40^. SA (*S*_BET_) (m^2^/g), the volume of monolayer (*V*_*m*_) (cm^3^) (STP), average pore diameter (MPD) (nm), total pore volume (*p*_*0*_/*p*_*0*_) (cm^3^/g) and energy constant (*C*) values of SDB, and SDBA were calculated using BET model. The mesoporous SA (*S*_*mes*_), microporous SA (*S*_*mi*_), the volume of mesoporous (*V*_*mes*_), and the volume of microporous (*V*_*mi*_) of SDB and SDBA biochars were assessed using the Barrett–Joyner–Halenda (BJH) model^41^. The computations were carried by with the BELSORP analysis program software. The form of the biochar's surface was examined using a scanning electron microscope (SEM) (Model QUANTA 250, Intertek MSG, TheWilton Centre, Redcar, UK, TS10 4RF). The functional groups on the surface of SDB and SDBA were investigated using Fourier Transform Infrared (FTIR) spectroscopy (Model VERTEX70, Germany) coupled with an ATR unit model V-100 in the 400–4000 cm^–1^ wavenumber range. X-ray diffractograms (XRD) was investigated using a Bruker Meas Srv (Model D2 PHASER, Germany) (D2-208219)/D2-2082019 diffractometer working at 30 kV and 10 mA with a 2*θ* range of 5–80 and a Cu tube (*λ* = 1.54). The SDT650-Simultaneous Thermal Analyzer instrument (TA Instruments Headquarters, 159 Lukens Drive, New Castle, DE 19720, USA) was used for thermal analyses with a temperature range of 50–1000 °C and a ramping temperature of 5 °C/min^42–44^. All adsorption experiments were repeated with a stander deviation of less than ± 2.7, and only the mean values were applied in tables, analyses, models and figures. This work used Excel 2013 and Origin 2019b programs for data analysis.

### Methods

#### Sawdust biochar (SDB) preparation

The collected wood sawdust (WSD) was thoroughly washed with tap water multiple times to remove dust and dried overnight at 105 °C. The WSD (200 g) was refluxed at 280 °C for 4 h in an 800 mL solution of 80% H_2_SO_4_, after which the mixture was filtered, washed with distilled water (DW) until the washing solution became neutral, and then washed with EtOH. The weight of the finished biochar product (92 g) was ascertained after oven drying at 105 °C. This process produced biochar which was named SDB^45,46^.

#### *Treatment of SDBO with NH*_*4*_*OH*

NH_4_OH (200 mL) was used to boil 50 g of SDB for 3 h in a fume hood under nitrogen, after which it was cooled, filtered, and washed with DW and EtOH. The solid biochar was branded as SDBA after drying overnight at 105 °C.

### Adsorption measurement for methylene blue dye

By dissolving 1.0 g of MB dye in 1000 mL of DW, a stock solution of the dye (1000 mg/L) was created, and this solution was diluted to achieve the necessary concentration for the adsorption investigation and the standard curve. To assess the adsorption capacity, thermodynamic, and kinetic properties of SDBA, which was created from SDB, batch adsorption experiments were used. A series of Erlenmeyer flasks (300 mL) containing 100 mL of various MB dye concentrations and varying biochar doses were shaken at 200 rpm for a predetermined period. 0.1 M HCl or M NaOH was used to adjust the sample pH to the required values. After separating the adsorbent from around 0.5 mL of the solution in the Erlenmeyer flask, the concentration of MB dye was measured at various times and in equilibrium. Spectrophotometry at λ_max_ 665 nm was used to assess the amount of MB dye present^47,48^. The adsorption capacities of MB dye at equilibrium (*q*_e_) were considered from Eq. (1):

1 $${q}_{e}=\frac{C_0-C_e}{W}\times V$$

where *q*_e_ is the MB dye amount per unit of absorbent at equilibrium (mg/g); *C*_0_ and *C*_e_ (mg/L^1^) are the starting and equilibrium MB dye concentrations in the liquid phase, respectively; *V* is the volume of the solution (L), and *W* is the SDBT mass in gram.

#### Solution pH impact

With 100 mL of 100 mg/L starting MB dye concentration and solution pH (2–12), the impact of pH on SDBT was examined. The pH_PZC_ has been studied for the SDBT following the literature-reported method^49^. 0.1 g of sample was added to 50 mL of 0.1 M NaNO_3_ solution with an initial pH range of 2 to 10, and the mixture was agitated for 24 h to calculate the SDBA pH_PZC_ value. The solution's equilibrium pH values were then measured using a pH metre after the sample was removed from it. The initial pH of the solution was plotted against the difference between starting and equilibrium pH. The pH_PZC_ value was recorded where the graph crosses the x-axis^49^.

#### The impact of the starting MB dye concentration, adsorbent dose, and contact time

The isotherm investigation for SDBA was carried out with varying starting MB dye solution concentrations (20 to 120 mg/L) and various masses of SDBT biochar (0.05 to 4.0 g/L). The isotherm investigation for SDBA was carried out with varying starting MB dye solution concentrations (20 to 120 mg/L) and various masses of SDBT biochar (0.05 to 4.0 g/L)^47,48^.

## Results and discussion

### SDB, and SDBA characterization

#### FTIR estimation of biochar surface functional groups

**Figure 1:**
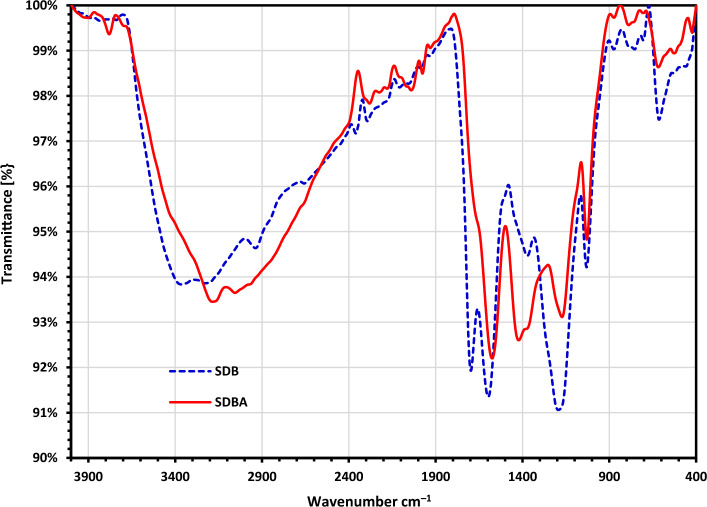
FTIR investigation of SDB and SDBA biochars before MB dye adsorption.

SDB, and SDBA underwent FTIR analysis to identify the functional groups on their surfaces and determine the impact of alteration on the disappearance or emergence of new functional groups. SDB, and SDBA biochars' FTIR analyses are displayed in Fig. 1. The peaks at 3363.03 and 2937.75 cm^–1^ showed the OH and alkyl groups' O–H and C–H vibration in SDB biochars. The COOH, C=C, –C–C– stretch (in-ring), and C=O are represented by the bands from 1698 to 1372 cm^–1^, which are referred to as "overtones," as band 1697.43 cm^–1^ in SDB^46,50^. The bands at 1193.5 and 1030.95 cm^−1^ in SDB are correlated to the C–O–H group vibrations^51–53^.

FTIR analysis of SDBA biochar showed broad adsorption peaks around 3185.1 and 3056.27 cm^−1^ inductive of the existence of the –OH and–NH groups in Sawdust-Biochar-NH_2_ (SDBA) (Fig. 1). These proved that NH_4_OH was successfully reacted with biochar to form nitrogen groups onto its surface. The band at 1575 cm^−1^ imply the C=C or C=N stretching vibration in SDBA biochars (Fig. 1). The new peak at 1422.2 cm^−1^ represents the C-NH functional group, indicating that the refluxing in 25% NH_4_OH solution have a substantial effect on the C–O or C–S functional group of biochar. The amination reaction leads to new functional group formation, as proved by the FT-IR spectra of SDBA. A New peak appeared between 1172.41 and 422.98 cm^−1^, proving the formation of new NH and N=C=O groups vibration (Fig. 1).

**Figure 2:**
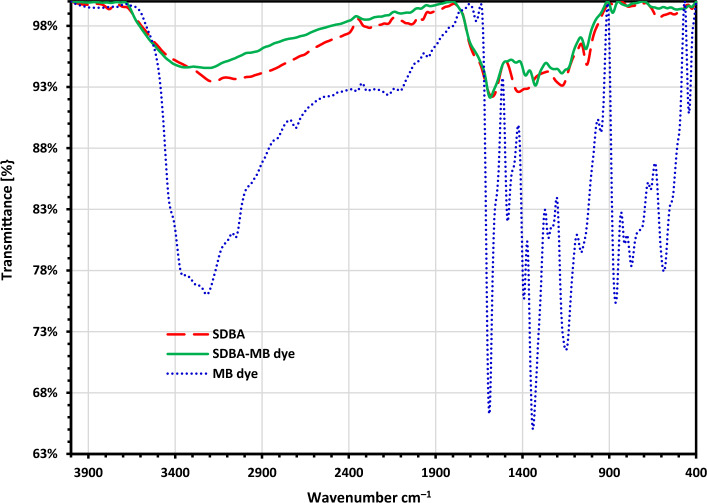
FTIR investigation of MB dye, SDBA, and SDBA-MB biochars after being contacted for 3 h with MB dye.

Figure 2 displays the FTIR spectrum of MB dye, SDBA, and SDBA-MB dye biochars. After the biochar under test was subjected to the MB dye adsorption method, it was found that all of the FTIR tests showed bands at 1636.44, 1591.44, 1435.03, 1357.29, 1218.9, 1179.34, and 1031.77 cm^–1^ that are related to the MB dye. Also a small shift occurred in the bands at 760.94, 665.60, 610.70, and 506.53 cm^–1^. These peaks proved the adsorption of methylene blue onto SDBA surface.

#### SDB and SDBA surfaces area analysis

**Figure 3:**
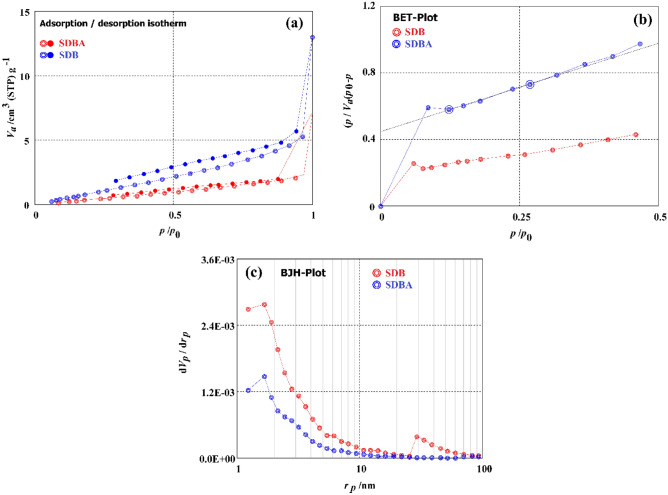
(**a**) Adsorption–desorption, (**b**) BET, (**c**) BJH investigation of SDB and SDBA.

N_2_ adsorption–desorption was used to examine how ammonia treatment affected the surface characteristics of wood sawdust biochar (SDB). To determine a particular feature of biochar surfaces, BET and BJH techniques were studied. The biochars' BET-specific surface area (SA) declined as SDB (6.61 m^2^/g) > SDBA (2.879 m^2^/g), as seen in Fig. 3. It should be highlighted that changes impact a particular surface area and that ammonia modification has a significant impact. The average pore diameter shrank in the following order: SDB (10.07 nm) >  > SDBA (6.29 nm), and ammonia modification had a significant influence on the reduction of pore diameter because of the addition of NH_2_ group may be close the pores. Mesoporous is the kind of pores in SDB, and SDBA biochars have total pore volumes of 16.664 × 10^–3^, and 5.521 × 10^–3^. BJH analysis results for SDB and SDBA biochars are shown in Fig. 3c and Table 1.

**Table 1:** BET and BJH models analyses of SDB and SDBA data.

Model	BET analysis data				BJH analysis data		
Biochar	SA (m^2^/g)	MPD (nm)	TPV (cm^3^/g)	*V*_m_ (cm^3^(STP)/g)	SA (m^2^/g)	*V*p (m^3^/g)	*r*p, peak (area) (nm)
SDB	6.608	10.074	0.016642	1.5182	7.4265	0.018113	1.66
SDBA	2.8769	6.2876	0.0045222	0.6610	3.2612	0.0051521	1.66

#### Morphological surface properties of SDB, SDBO, and SDBT

**Figure 4:**
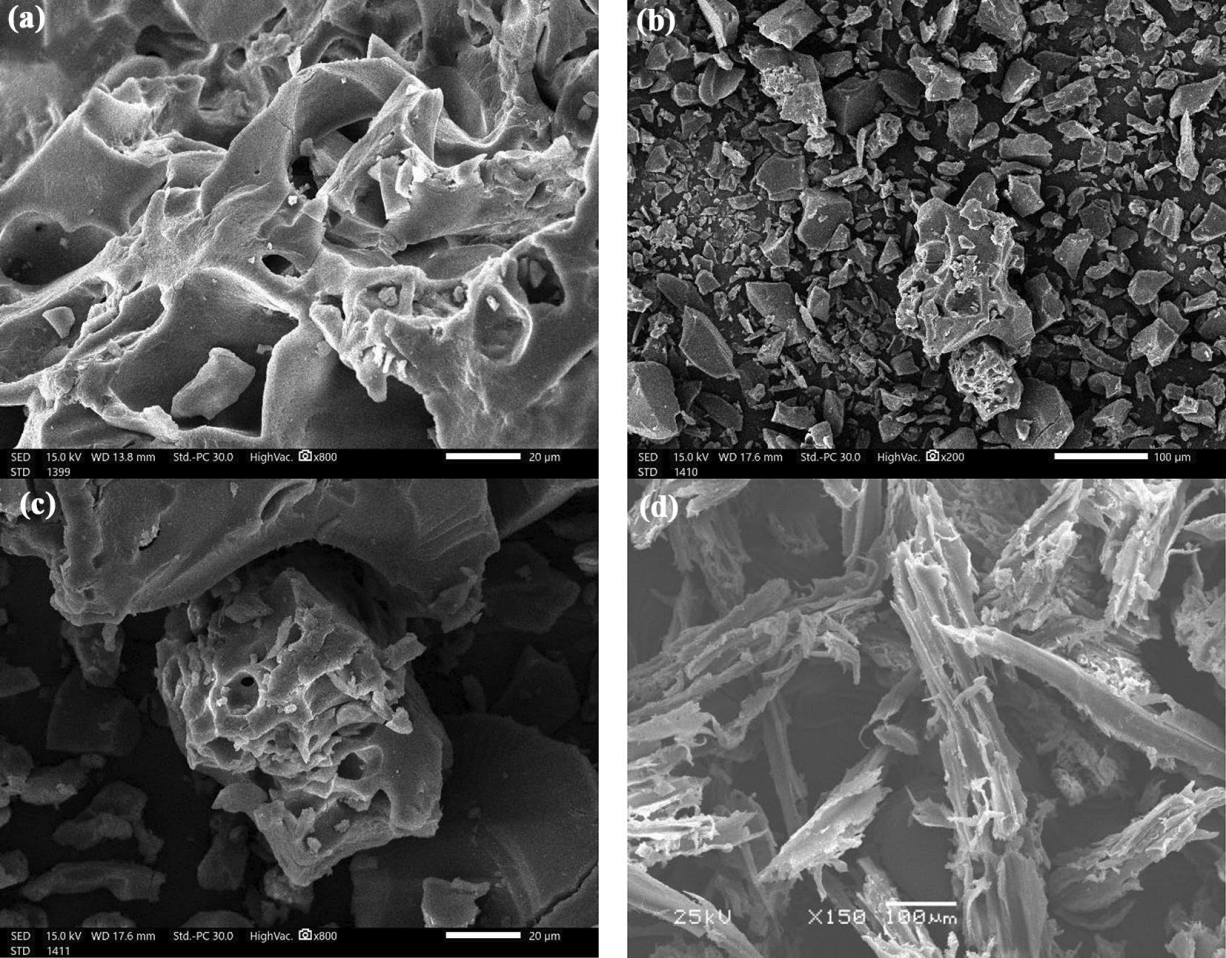
Scan electron microscope investigation of (**a**) SDB magnified for 800x, (**b**) SDBA magnified for ×200, (**c**) SDBA magnified for ×800 biochars, and (**d**) Raw SD material (RSD) magnified for 150x.

Figure 4 shows the results of a scan electron microscopy (SEM) analysis of the surface morphology of sawdust raw material (RSD), SDB and SDBA. The SDB biochar, as illustrated in Fig. 4a, looks clean and devoid of any impurities or particulates, and no damage to the SDB's pores due to the dehydration process with 80% H_2_SO_4_ was noticed. As a result of amination surface modification, Fig. 4b,c depicts the SDBA biochar as having a few tiny holes corresponding to the SDB biochar. This validates our earlier discovery that ammonia treatment of biochar in water resulted in pore blockage, which reduced surface area^51–53^. It is apparent that the ammonia reflux in water caused the pore to be blocked and the SA of the SDBA to diminish. During this process, the oxygen and sulfur groups were replaced with NH_2_ groups, decreasing the surface area of SDBA (2.8769 m^2^/g) lower than SDB (6.608 m^2^/g). No holes were seen in the RSD's SEM picture depicted in Fig. 4d.

#### SDB, and SDBA elemental analysis

The chemical makeup of SDBand SDBA biochars was examined using an Energy Dispersive X-ray spectrometer (EDX). Table 2 displays the data of the analysis of the elemental percentages of SDB, and SDBA biochars and illustrates the lack of nitrogen peak prior to ammonium hydroxide modification. The EDX analysis of SDBA biochar revealed that 7.63% of the sample weight was nitrogen, and 2.23% was sulfur. The EDX analysis proved that most of the sulfur atoms in SDB were replaced by nitrogen atoms.

**Table 2:** EDX investigation results of SDB and SDBA biochars.

Biochar	SDB		SDBA	
Elements	Wt%	At%	Wt%	At%
Carbon	48.23	56.07	57.49	64.32
Nitrogen	ND	ND	7.63	7.32
Oxygen	28.15	26.98	32.65	27.42
Sulfur	23.62	16.95	2.23	0.94

NA: not detected.

#### SDB and SDBA thermal characterization

**Figure 5:**
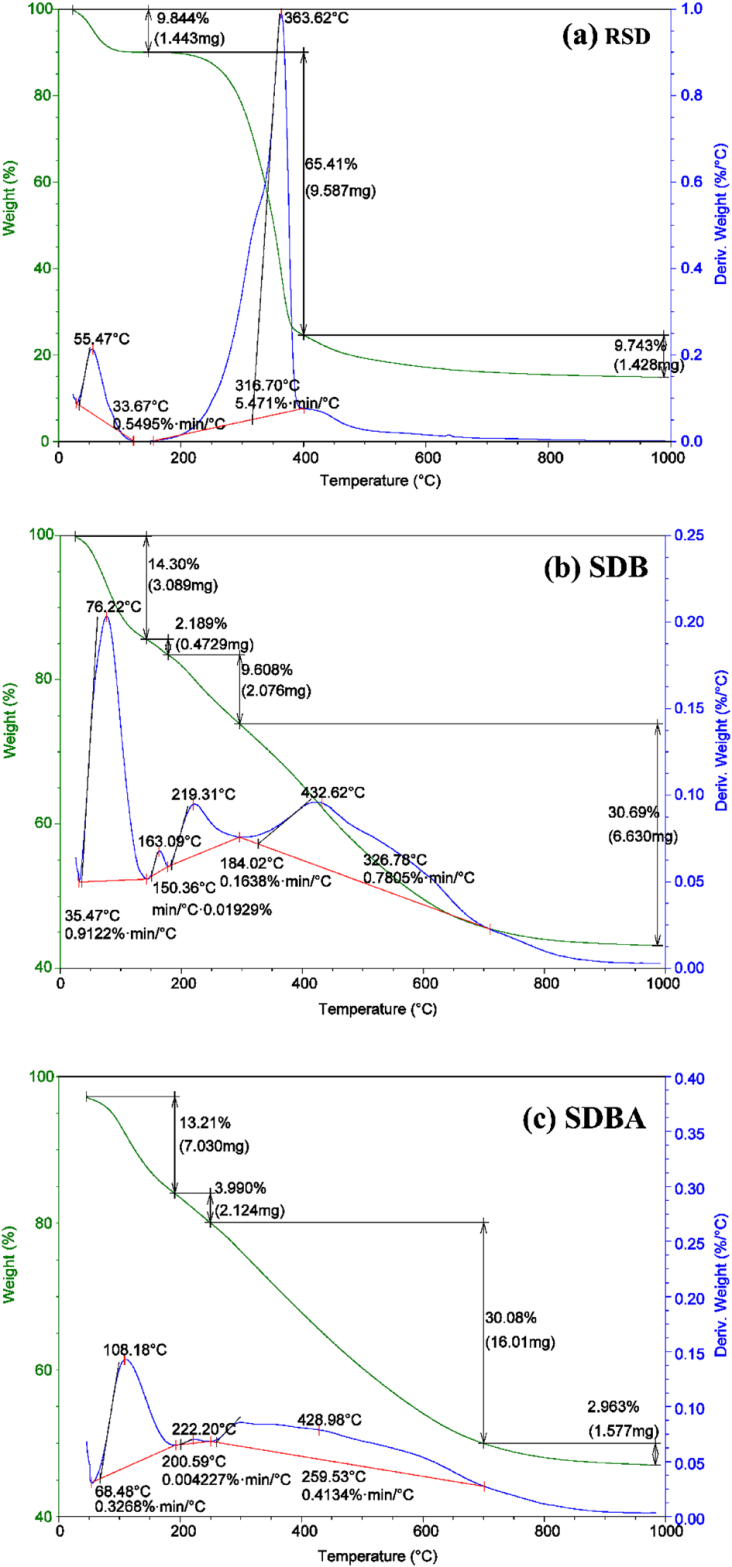
TGAand DTA investigation of (**a**) RSD material, (**b**) SDB, and (**c**) SDBA biochars.

Figure 5 depicts the TGA breakdown of unprocessed RSD, SDB and SDBA biochars. With respect to RSD, the first step of breakdown (Fig. 5a) occurs at temperatures between 50 and 140 °C and entails the loss of moisture and surface-bound water. This stage generally causes a mass loss of 9.84%. The breakdown of organic content in the biochar occurs in the second stage at temperatures between 140 and 400 °C, which results in a significant mass loss of around 65.41% of the biochar's original weight. Hemicellulose, cellulose, and lignin degradation in the feedstock material is responsible for this step. The third stage, which takes place between 400 and 1000 °C, involves the breakdown of the remaining organic materials, including aromatic compounds, and causes a weight loss of around 9.74%. It is thought that the breakdown of more sophisticated organic molecules in the feedstock is what causes this stage. About 85% of the RSD sample mass was comprised of the three mass losses. The moisture and surface-bound water contained in the RSD sample were reflected by two significant peaks in the DTA analysis at 55.47 °C, and the substantial weight loss was indicated by a high peak at 363.62 °C (Fig. 5a). With a weight loss of around 14.30%, the first stage of breakdown takes place in the SDB biochar sample (Fig. 5b) at temperatures between 50 and 140 °C. The second phase involves temperatures between 140 and 175 °C and a weight loss of about 2.20%. Temperatures between 175 to 300 °C are used in the third breakdown stage, with a weight loss of around 9.61%. Temperatures between 300 and 1000 °C are used in the fourth breakdown process, with an estimated weight loss of 30.69% (Fig. 5b). About 56.8% of the SDB sample mass was comprised of the four mass losses. The moisture and surface-bound water contained in the sample were represented by four significant peaks in the DTA analysis of the SDB sample at 76.22 °C, and a tiny peak representing a modest weight loss was seen at 163.09 °C (Fig. 5b). The third and fourth peaks occurred at 219.31 and 432.62 °C represented 41.21% mass loss of the SDB sample. The SDBA biochar sample analysis shows four weight loss positions with total mass loss representing 50.24% of the sample mass (Fig. 5c). At temperatures between 50 and 170 °C, the first breakdown stage takes place and results in a weight loss of around 13.21%. 3.99% of the weight was lost in the second stage at temperatures between 170 and 250 °C, while 30.08% was lost in the third decomposition process as the highest mass loss at temperatures between 250 and 700 °C (Fig. 5c) which involves the decomposition of cellulose and lignin in the biochar. The fourth mass loss occurred from 700 to 1000 °C as 2.96% of the total sample mass. The DTA analysis of SDBA sample represented three significant peaks at 108.18 °C for moisture and surface-bound water existing in the sample and at 222.20 °C as a moderate peak represented a slight weight loss, and the third peak was presented at 428.98 °C showing the significant mass loss percent (Fig. 5c).

#### XRD characterization of SDB and SDBA

**Figure 6:**
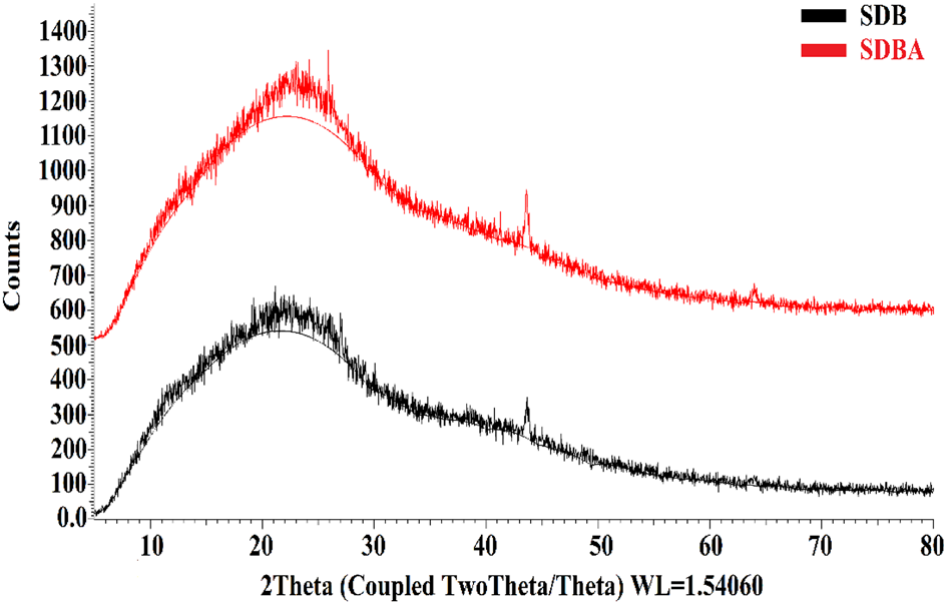
XRD analysis of prepared SDB and SDBA biochars.

Figure 6 shows the XRD of the SDB and SDBA biochars. The wide peak at 2*θ* = 10–30 is designated as the C (002) diffraction peak, which denotes an amorphous carbon structure with aromatic sheets arranged randomly. There are two distinct peaks for SDB at 2 = 27 and 43.6. Within SDBA's SDB structure, two sharp peaks at 2*θ* = 25.89 and 43.62 correspond to inorganic compounds like quartz^54,55^.

### Adsorption of MB dye on SDBT

**Figure 7:**
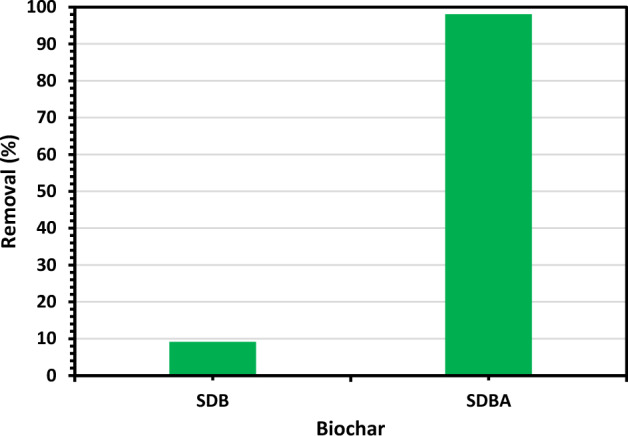
Test of MB dye removal by SDB and SDBA biochars (MB dye *C*_0_: 120 mg/L and 1.0 g/L biochar dose at 25 ± 2 °C.

The removal of MB dye by SDB, and SDBA was tested to select which biochar has the highest tendency to absorb MB dye from water. Figure 7 shows the adsorption test of MB dye by prepared SDB, and SDBA biochars. As seen from Fig. 7, SDB shows a deficient removal % for MB dye, while the aminated biochar SDBA showed a high ability to absorb MB dye from its water solution giving over 98% removal.

### Adsorption of MB dye on SDBA

#### Effect of pH

**Figure 8:**
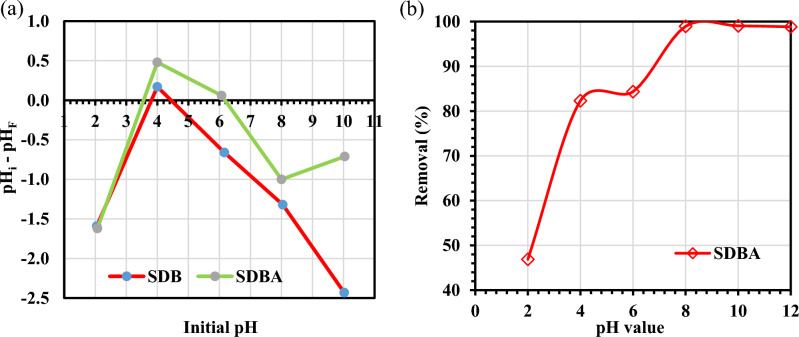
(**a**) pH_ZPC_ graph of SDB and SDBA, (**b**) effect of solution pH on the MB dye removal % by SDBA (*C*_0_ of MB = 20 mg/L, SDBA dose = 1 g/L at 25 ± 2 °C).

The initial pH of the solution was plotted against the difference between the initial and equilibrium pH (Fig. 8a). The pH_PZC_ value was documented at the location where the graph crosses the x-axis. The pH_PZC_ value for SDB and SDBA crossed the x-axis at two points for both biochars. The pH_PZC_ values for SDBA were 3.5 and 6.2, which are in the acidic range. Also, the pH_PZC_ values for SDB were 3.8 and 4.4, which are in the acidic range too. To predict the adsorption process, the pH is a vital operational parameter that affects the surface charges of the SDBA and interfacial transport phenomena^56^. Moreover, the pH value can impact the metal ionization or dissociation level, the chemical makeup of dyes, and the surface characteristics of adsorbents^57^. MB dye removal was studied at different pH ranges between 2 and 12. Figure 8b shows the adsorption of MB dye as a function of solution pH. As indicated in Fig. 8b, the highest removal efficiency occurred at pH 8. At acidic pH circumstances, the removal efficiency dropped, while it stayed nearly constant in basic settings. MB removal was up to 98.94% at a solution pH of 8. The pH of the medium has an impact on the binding sites on the SDBA surface. Functional groups on the adsorbent surface become protonated in a low-pH environment. The positively charged adsorbent surface's capacity to interact with the positively charged cationic dye MB is diminished due to electrostatic repulsion. However, the negatively charged functional groups on the adsorbent can interact electrostatically to combine with the positively charged MB dye when the pH of the solution increases, and the positively charged groups on the SDBA deprotonate.

As a result, using an adsorbent greatly increases the rate of MB elimination. The outcomes demonstrate that one of the MB adsorption mechanisms is electrostatic contact. Similar results were reported in the works of Ait Ahsaine et al.^22^ and You et al.^6^. The surface functional groups and biochar composition can affect the occurrence of two pH_PZC_. If the concentrations of these surface functional groups are high enough and their dissociation constants are sufficiently different, biochar with multiple surface functional groups such as carboxylic acids (–COOH) and hydroxyl groups (–OH) or amines (–NH_2_) and hydroxyl groups (–OH) can potentially display two pH_PZC_. The biochar's structure, in particular its pore structure and surface area, can affect the presence of two pH_PZC_. The overall surface charge of the substance may change depending on how accessible the surface functional groups are to ions in solution due to the pore structure of the biochar. Due to more surface functional groups, biochar with a high concentration of micropores may have a more negatively charged surface, which could lead to a lower pH_PZC_. On the other hand, biochar with a large proportion of macropores may have a surface that is less negatively charged because there are fewer surface functional groups present, which could lead to a greater pH_PZC_.

#### Impact of contact time

**Figure 9:**
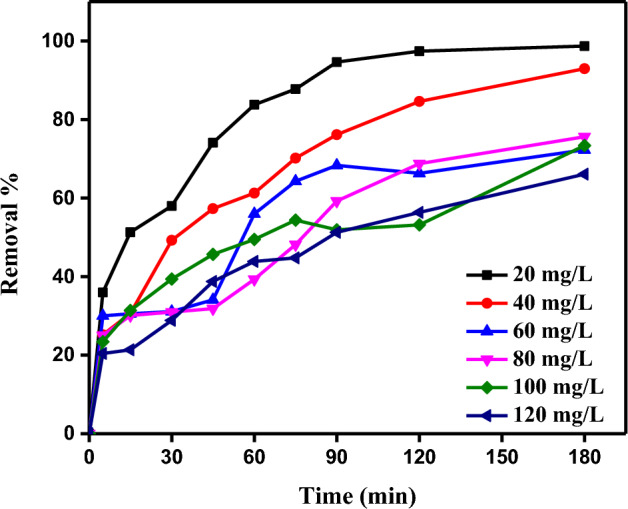
The MB dye removal for 180 min by SDBA (*C*_0_ of MB dye (20–120 mg/L), SDBA mass 0.5 g/L, Temp. 25 ± 2 °C).

The Methylene Blue (MB) dye adsorption tests were carried out at various contact durations (from 0 to 180 min.) and initial MB concentrations (20 to 120 ppm) to look into the adsorption kinetics. No extra compounds were used in any of the trials. At 298 K, 0.5 g/L, and 180 min of contact time, it was examined how the contact time affected the adsorption efficiency. Figure 9 shows how the initial MB concentration affected the adsorption efficiency. When the contact time rose, the removal increased as well, and after some time, the adsorption reached equilibrium. Occasional deviations were observed in the curves of different initial concentrations. At MB concentrations of 20 and 120 mg/L, the highest adsorption efficiency was found to be 98.69% and 66.09%, respectively. At low starting MB dye concentrations, the results were explained by a sufficient number of active sites, but the persistent active sites had trouble tying up the rising MB molecules from the bulk phase. Additionally, a higher dye concentration may also result in a higher dye cation repulsion, which would reduce the effectiveness of the adsorption^58^. Increasing the starting MB dye concentration increases the MB dye's capacity to adsorb because it gives a more potent driving force to overcome the mass transfer barrier between the aqueous phase and SDBA.

#### Impact of starting MB dye concentration

**Figure 10:**
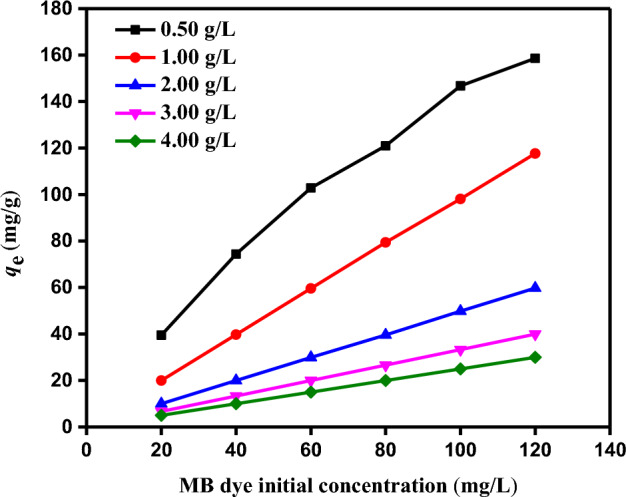
The impact of MB dye starting concentration (20–120 mg/L) using SDBA mass (0.5–4.0 g/L) on *q*_*e*_ (mg/g) (Temp. 25 ± 2 °C).

The starting dye concentration affected the adsorption of dyes. Figure 10 depicts the relationship between time and the SDBA's MB dye adsorption capabilities. Using SDBA dosages of 0.5–4.0 g/L at constant temperatures of 25 °C and pH 8.0, the effect of starting dye concentration was examined on MB dye solutions with known starting concentrations of 20–120 mg/L. According to Fig. 10, the adsorption capability steadily rises as the MB dye concentration rises for all different dosages of SDBA adsorbent. Initial MB dye concentrations (20, 40, 60, 80, 100 and 120 mg/L) have *q*_e_ vary from 39.47 to 158.62 mg/g for 0.5 g/L, 19.97 to 117.67 mg/g for 1 g/L, 9.99 to 59.76 mg/g for 2 g/L, 6.66 to 39.92 mg/g for 3 g/L, and 4.99 to 29.95 mg/g for 4 g/L SDBA mass. A similar trend was seen in El Nemr et al.'s study^59^ on the Acid Yellow 11 dye adsorption.

#### Impact of SDBA mass on MB dye adsorption

**Figure 11:**
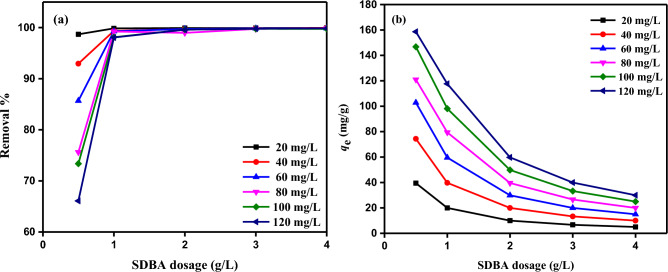
The impact of SDBA different masses (0.5–4.0 g/L) of different starting MB dye concentrations (20–120 mg/L) (**a**) on removal %; (**b**) on *q*_*e*_ (mg/g), (Temp. = 25 ± 2 °C).

The impact of SDBA dose on the removal of MB dye at various starting MB dye concentrations is depicted in Fig. 11. It was discovered that when dosage increased up to 1.0 g/L, the MB dye removal effectiveness rose significantly (Fig. 11a). Increasing the SDBA mass from 1.0 to 4.0 g/L did not cause a significant change in MB dye removal and remained constant. The elimination efficiency of 120 mg/L MB dye starting concentration rose from 66.09 to 99.84% for a rise in SDBA mass from 0.5 to 4.0 g/L. Also, it was discovered that for an initial concentration of 120 mg/L MB dye, the *q*_*e*_ fell from 158.62 to 29.95 mg/g with an increase in the dosage of the SDBA adsorbent (Fig. 11b). The starting dye concentration, the available sorption surface, and the adsorption sites act as the driving force to overcome the obstacle to the mass transfer of dye between the solid and aqueous phases. The interaction between the MB dye and adsorbent is improved by the increase in adsorption sites and the accessible sorption surface, which increases the effectiveness of MB dye removal. Because there are fewer MB dye molecules present at each adsorption site, there is a "less intense driving force" that favors adsorption to the solid surface, which results in a reduction in the quantity of dye adsorbed per unit mass of adsorbent at equilibrium (*q*_e_) at each site.

### Adsorption isotherms

The adsorption isotherms indicate how the SDBA molecules are distributed between the solid and liquid phases after the system reaches equilibrium^60^. Designing adsorption systems requires analysis of the equilibrium data. Three adsorption isotherms models, including the Langmuir (LIM)^61^, Freundlich (FIM)^62^, and Tempkin (TIM)^63^ Isotherm Models (IMs), were used to characterize the equilibrium data from this investigation. As two-parameter models, the LIM and FIM include details on the adsorption capacity and constants relating to the activation energy^64^. LIM presupposes that the adsorption process occurs on a homogeneous surface, where the molecules create a monolayer of adsorbate on the material's surface, saturating the pores and inhibiting transmigration^65^. FIM is an empirical equation considering adsorption on diverse surfaces^62^. If the adsorption process is physical (*n*_*F*_ > 1), chemical (*n*_*F*_ < 1), or linear (*n*_*F*_ = 1), it is indicated by the heterogeneity factor (*n*_*F*_). Moreover, a value of 1/*n*_*F*_ > 1 suggests cooperative adsorption, whereas 1/*n*_*F*_ < 1 indicates a conventional LIM^66^. TIM assumed that the heat of adsorption on adsorbate molecules linearly reduces when surface coverage rises due to adsorbate-adsorbent interaction^63^.

**Figure 12:**
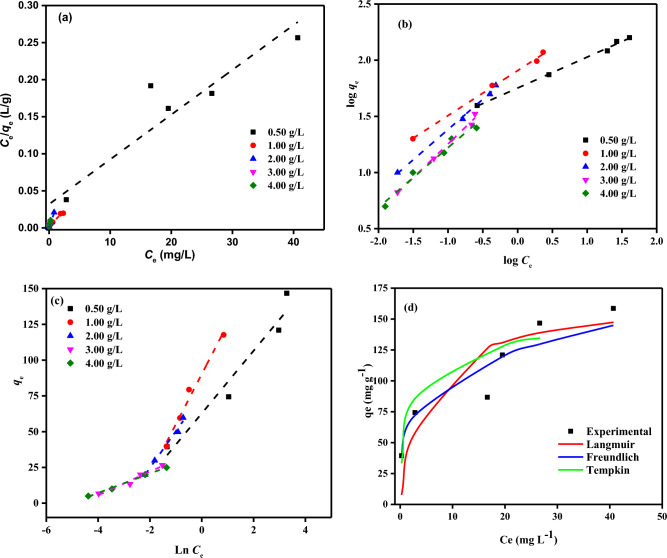
(**a**) LIM (**b**) FIM (**c**) TIM profiles for *C*_*0*_ of MB dye (20–120 mg/L) on SDBA doses (0.5–4.0 g/L) at (25 °C), (**d**) Comparison of measured and modelled isotherm profiles for MB dye (*C*_0_: 20–120 mg/L, SDBA mass: 0.5 g/L, Temp: 25 ± 2 °C.

The values *R*^2^ calculated from linear regression of the least squares fit statistic were used to determine the suitability and applicability of the IMs to the equilibrium data. According to the three models in Fig. 12a–c, the MB dye solution's adsorption isotherm on SDBA was as expected. In Fig. 12d, these three models were compared with the experimental data. Table 3 contains the parameters for fitting these isotherm models. The FIM (*R*^2^ > 0.972) and LIM (*R*^2^ > 0.965) models produced the best *R*^2^ match for the adsorption data. Freundlich constants *i.e.* adsorption rate, *n*_*F*_, and adsorption capacity, *K*_*F*_ are determined from this plot, which is in the range of 0.01–3.85 and 54.89–82.89 mg/g, respectively. The *K*_*L*_ of < 1 and the *n*_*F*_ value of > 1 obtained for LIM and FIM indicate that the adsorption is favorable. Moreover, the FIM estimated that the *Q*_m_ was 643.74 mg/g. According to the Literature, the maximal SDBA capacity for MB dye is relatively high. The adsorption data were well-fit by the Temkin isotherm (*R*^2^ > 0.948). Hence, electrostatic contact is one of the processes involved in the adsorption of MB dye onto SDBA. The linear relationship between *q*_*e*_ and ln* C*_*e*_ illustrated in Fig. 12c can be used to compute the TIM parameters (*A*_*T*_ and *B*_*T*_). The slope of this graph gives *A*_*T*_ (g/L), and the intersection gives *B*_*T*_. The adsorption of MB dye by SDBA depends heavily on the heat of adsorption (*B*_*T*_), which is released due to the interaction between the adsorbate and adsorbent. When the collected data were evaluated, it became clear that physisorption was used to achieve the adsorption because the adsorption temperatures were at such low values.

**Table 3:** IM investigation data of MB dye adsorption by SDBA (MB (20–120 mg/L), SDBA masses (0.5–4.0 g/L), Temp. (25 ± 2 °C)).

IM	Parameters	SDBA adsorbent doses (g/L)				
		0.50	1.00	2.00	3.00	4.00
LIM	*Q*_*m*_ (mg/g)	166.67	129.82	48.30	46.94	31.74
	*K*_*L*_ × 10^3^	0.19	2.41	14.78	7.61	13.60
	*R*^2^	0.980	0.965	0.971	0.999	0.999
FIM	*1/n*_*F*_	0.26	0.41	0.55	0.64	0.57
	*Qm* (mg/g)	127.53	308.52	471.38	643.74	403.45
	*K*_*F*_ (mg^1–1/n^ L^1/n^ g^–1^)	54.89	81.75	80.78	82.89	64.85
	*R*^2^	0.995	0.997	0.997	0.992	0.972
TIM	*A*_*T*_	17.64	13.07	19.30	106.70	156.02
	*B*_*T*_	21.87	35.04	25.64	8.25	6.78
	R^2^	0.948	0.984	0.988	0.965	0.996

The impact of primary function OH in a basic pH medium on the mechanism of dye adsorption by SDBA is to produce an alkaline environment that facilitates the adsorption process. The surface of biochar becomes negatively charged in an alkaline environment, attracting positively charged dye molecules. Additionally, the carboxylic and phenolic functional groups on the surface of the biochar react with the OH ions in the solution to create negatively charged sites that can further adsorb positively charged dye molecules. A basic pH makes dye molecules more soluble, making it more straightforward to diffuse into the biochar pores and bind to the adsorption sites. This makes SDBA an excellent way to remove color from industrial wastewater since the basic pH is essential in facilitating dye molecules' adsorption onto biochar.

The most significant mechanism entails the electrostatic interaction-mediated adsorption of ionizable organic molecules to the positively charged surface of the biochar^67^. The pH and ionic strength of the aqueous solution determine how well it attracts or repels contaminants^68,69^. Mukherjee et al.^70^ examined the impact of pH on the electrostatic interaction between organic pollutants and biochar surface, finding that a positive charge was present at low pH values while a negative charge was present at high pH levels. This suggests that the pH of the solution or effluent controls the net charge of the biochar's surface. The aqueous solution's ionic strength impacts how electrostatically the organic pollutants interact with the biochar. Inyang et al.'s^71^ study on the removal of methylene blue using carbon nanotube- and bagasse-based biochar demonstrated that increasing the sorbate solution's ionic strength from 0.01 to 0.1 M NaCl decreased the amount of methylene blue that was adsorbed, from 4.5 to 3 mg/g. The increase in the repellent electrostatic interaction between the sorbent and the sorbate was the reason for this.

### Error function investigation for best-fit IMs

To choose the best IMs for the adsorption of MB dye to SDBA adsorbent, *R*^2^ of FIM, LIM, and TIM isotherm models were evaluated with experimental equilibrium data. Comparing the error functions is another way to choose the best model. These can be listed as a hybrid error function (HYBRID), average percent errors (APE), Marquardt's percent standard deviation (MPSD), Chi-square error (X^2^), the sum of absolute errors (EABS) and root mean square errors (RMS)^72^. Since the models with values closest to zero will be the most suitable models, the LIM and FIM fit the experimental data rather well when all error functions are compared. Error function values of LIM, FIM and TIM models are given in Table 4.

**Table 4:** Some error function values of IMs used in the adsorption of MB dye on SDBA.

IM	Hybrid	RMS	APE (%)	X^2^	MPSD	EABS
LIM	2.792	0.436	0.087	0.642	0.454	0.087
FIM	2.939	0.487	0.081	0.676	0.508	27.758
TIM	28.379	1.646	0.329	6.527	1.716	79.315

### Adsorption kinetic studies

**Figure 13:**
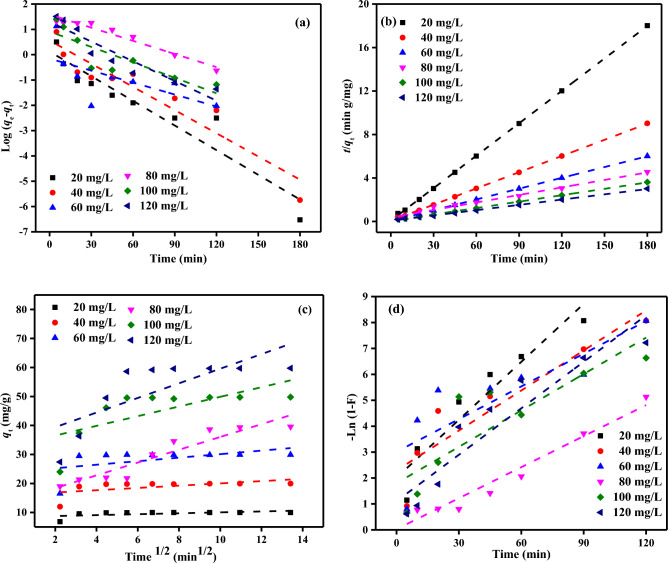
(**a**) PFOM (**b**) PSOM (**c**) IPDM (**d**) FDM of adsorption of MB dye by SDBA (*C*_0_ = (20–120 mg/L), Adsorbent dose (2.0 g/L), Temp. 25 ± 2 °C).

To comprehend the dynamics of the reaction in terms of the rate constant's order, kinetic studies are crucial. The data for the adsorption system's adsorption kinetics were examined by the non-linear fitting of four alternative kinetic models: the pseudo-first-order (PFOM), pseudo-second-order (PSOM), intra-particle diffusion model (IPDM), and film diffusion model (FDM), which are all depicted in Fig. 13. The kinetic parameters obtained from the PFOM, PSOM, IPDM and FDM are listed in Tables 5, 6.

**Table 5:** PFOM and PSOM results of adsorption of MB dye by SDBA (*C*_0_: (20–120 mg/L), SDBA masses (0.5–4.0 g/L), 25 ± 2 °C.

Parameter			PFOM			PSOM		
SDBA (g/L)	MB dye (ppm)	*q*_*e*_ (exp.)	*q*_*e*_ (calc.)	*k*_*1*_ × 10^3^	*R*^2^	*q*_*e*_ (calc.)	*k*_*2*_ × 10^3^	*R*^2^
0.50	20	39.48	16.88	0.25	0.239	42.37	3.09	0.999
	40	74.35	458.46	0.91	0.253	81.30	0.48	0.992
	60	102.82	19.93	0.06	0.013	97.09	0.32	0.972
	80	120.99	443.81	0.67	0.208	136.99	0.21	0.941
	100	146.75	443.81	0.75	0.188	172.41	0.19	0.979
	120	158.62	578.10	0.79	0.205	142.86	0.28	0.950
1.00	20	19.97	12.17	0.08	0.880	20.24	27.89	1.000
	40	39.74	19.59	0.04	0.981	41.15	5.09	1.000
	60	59.57	50.86	0.04	0.978	64.52	0.66	0.996
	80	79.39	66.68	0.05	0.961	84.03	0.65	0.997
	100	98.11	735.19	0.09	0.726	107.53	0.19	0.995
	120	117.67	88.72	0.02	0.973	129.87	0.13	0.990
2.00	20	9.99	1.33	0.07	0.903	10.05	151.84	1.000
	40	19.94	3.63	0.07	0.895	20.12	29.34	1.000
	60	29.92	1.43	0.04	0.407	30.21	3.46	1.000
	80	39.58	38.59	0.04	0.954	43.48	4.06	0.986
	100	49.80	8.24	0.05	0.736	43.48	8.04	1.000
	120	59.76	19.75	0.06	0.878	61.73	3.99	0.999
3.00	20	6.66	3.40	0.04	0.678	6.68	262.37	1.000
	40	13.31	1.86	0.07	0.929	13.37	100.81	1.000
	60	19.97	3.11	0.03	0.400	20.04	19.73	1.000
	80	26.59	1.99	0.07	0.884	26.67	14.71	1.000
	100	33.25	1.32	0.04	0.292	33.44	13.97	1.000
	120	39.92	1.17	0.05	0.717	40.00	9.77	1.000
4.00	20	5.00	2.07	0.07	0.811	5.01	458.92	1.000
	40	9.99	4.22	0.04	0.699	10.02	254.73	1.000
	60	14.98	2.89	0.04	0.648	15.02	96.01	1.000
	80	19.97	1.22	0.05	0.797	20.04	46.72	1.000
	100	24.94	5.69	0.02	0.613	24.94	15.89	1.000
	120	29.95	1.20	0.05	0.736	30.12	10.89	1.000

**Table 6:** IPDM and FDM results of MB dye adsorption by SDBA (*C*_0_: (20–120 mg/L), SDBA masses (0.5–4.0 g/L), Temp. (25 °C)).

Parameter		IPDM			FDM	
SDBA (g/L)	MB dye (mg/L)	*К*_*dif*_	*C*	*R*^2^	*К*_*FD*_	*R*^2^
0.50	20	2.79	11.19	0.939	0.037	0.991
	40	4.85	14.35	0.950	0.021	0.972
	60	5.14	20.86	0.907	0.027	0.936
	80	7.84	17.29	0.962	0.018	0.973
	100	7.47	38.21	0.920	0.015	0.826
	120	9.99	28.48	0.973	0.018	0.955
1.00	20	0.53	14.69	0.472	0.066	0.924
	40	2.73	17.12	0.878	0.054	0.978
	60	4.36	15.61	0.929	0.039	0.991
	80	4.36	35.77	0.889	0.053	0.985
	100	8.03	17.56	0.979	0.029	0.975
	120	8.57	17.66	0.990	0.022	0.976
2.00	20	0.16	8.44	0.319	0.107	0.937
	40	0.39	16.10	0.313	0.078	0.891
	60	0.61	24.03	0.258	0.079	0.838
	80	2.21	13.99	0.889	0.040	0.982
	100	1.67	33.19	0.487	0.070	0.883
	120	2.52	34.37	0.587	0.074	0.945
3.00	20	0.06	6.01	0.310	0.098	0.930
	40	0.15	11.87	0.316	0.088	0.899
	60	0.22	17.82	0.262	0.084	0.751
	80	0.29	23.78	0.277	0.102	0.798
	100	0.55	27.89	0.448	0.088	0.742
	120	0.47	35.35	0.285	0.091	0.936
4.00	20	0.03	4.64	0.309	0.081	0.805
	40	0.08	9.25	0.287	0.084	0.806
	60	0.12	13.82	0.271	0.085	0.801
	80	0.18	18.23	0.285	0.088	0.957
	100	0.03	24.67	0.446	0.086	0.730
	120	0.41	26.01	0.265	0.097	0.846

The experimental data could be better suited to the PFOM, as seen by the poor linear regression correlation coefficients *R*^2^ (0.013–0.981), as shown in Fig. 13a. According to Table 5; the PFOM has poor *R*^2^ values. According to Table 5, the PFOM has poor *R*^2^ values. This table also reveals a significant discrepancy between the experimental and predicted adsorption capacities. This illustrates a poor match of the PFOM to the experimental data.

According to the PSOM, the adsorption process is the phase that limits the pace of the reaction. The rate-limiting mechanism might be a chemical reaction in which the SDBA and dyestuffs exchange or share electrons to change the valence forces^73,74^. The experimental data are pretty well suited to the PSOM, as seen by the good linear regression correlation coefficients *R*^2^ (0.941–0.981), as shown in Fig. 13b. The results of Table 5 demonstrate that PSOM more accurately captures the adsorption kinetics data, and the computed *q*_*e*_ values match the experimental *q*_*e*_ values. It is clear that as starting MB dye concentrations rise, the value of the rate constant *k*_*2*_ generally falls (Table 5). This may be explained by the fierce competition for concentrated sorption surface locations, which increases sorption rates. This shows the MB adsorption on SDBA to have second-order kinetics. These findings concur with those provided by Hameed and Daud^75^.

The pattern of intraparticle diffusion^76^ was also examined as the adsorbate was transported from the MB dye solution to the SDBA surface. According to the hypothesis by Weber and Morris^76^, the intraparticle diffusion step governs the adsorption when the dashed lines depicted in the graph of *q*_*t*_ and root time (*t*) in Fig. 13c pass through the origin. As seen in Table 6, the IDM's *C* values do not equal zero at any of the initial MB concentrations that were investigated, demonstrating that intraparticle diffusion is not the only factor influencing the MB dye adsorption process. Consequently, a complicated interaction between the boundary layer and intraparticle diffusion may be the rate-limiting step. Additionally, based on this graph, it is assumed that film diffusion regulates the rate of the adsorption process if the dotted lines do not intersect the origin. The IPDM is used to explain solid–liquid adsorption solute transfer. Three processes are involved in the deposition of the adsorbate on the adsorbent. The first is the transportation of the ions or molecules in the solution to the adsorbent surface through the liquid layer. The ions and molecules on the surface of the adsorbent subsequently diffuse within the adsorbent. The last stage involves chemical reactions involving the active groups of the adsorbent. The slower of these three steps, which occur at various rates, determines the adsorption rate.

### Adsorption mechanism of MB dye by SDBA

**Figure 14:**
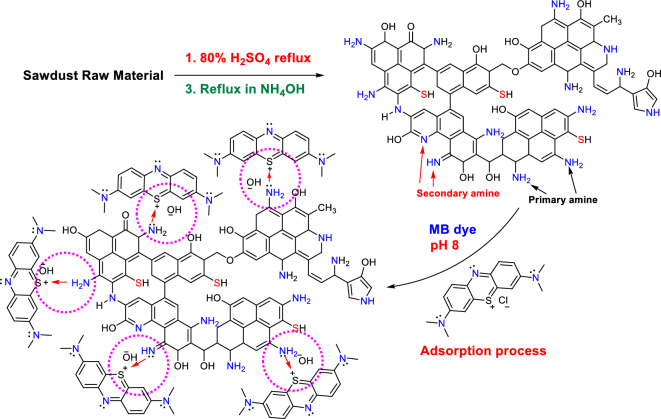
Probable mechanism for the MB dye adsorption onto the SDBA biochar.

The probable mechanism for the adsorption of the MB dye ions by SDBA was explained in Fig. 14. After the dehydration of sawdust raw material with 80% H_2_SO_4_ and treatment with ammonia at reflux; many functional groups were formed on the adsorbent (SDBA) surface like C=N, amide N–H, hydroxyl O–H, C–N and SH groups as found from FTIR analysis. The MB dye ions adsorption mechanism in a base medium (pH 8) may be achieved via physical interaction due to electrostatic interaction between the nitrogen lone pair on the SDBA surface and the positive charge on the sulfur atom of the MB dye. The surface charge became positive, which attracted the hydroxyl ions from the base medium due to the basic pH 8. Moreover, the pH of the solution also affects the adsorption capacity of organic contaminants of industrial wastewater^67^. The adsorption of textile dyes in wastewater using biochar produced from food waste was investigated^77^. An alkaline pH enhanced the adsorption of dyes, and it attributed to the high interaction between the negatively charged sites on the biochar’s surface with the positively charged dyes. In contrast, at pH 3, its efficiency in adsorbing organic dye decreased because of the existence of extra H^+^ that compete with the positive charge of the dye. Tsai and Chen^78^ and Xu et al.^37^ reported similar observations on the effect of pH on the adsorption capacity of biochar. Thus, the pH of the solution affects the adsorption capacity of biochar for organic and inorganic pollutants from water by altering the charged sites.

### Comparison with the findings from the literature

The obtained adsorption capacity is indicated in Table 7. The *Q*_m_ of MB dye uptake per unit mass of SDBA is 643.74 mg/g based on the Freundlich equilibrium model and is suitable for other adsorbents made from other precursors to purge MB dye. The outcomes demonstrated that the SDBA is a remarkable adsorbent for the cationic dye adsorption process.

**Table 7:** Comparison of the MB dye adsorption capabilities of some adsorbents.

Adsorbent name	*Q*_m_ (mg/g)	Ref.
Albizia lebbeck seed pods	328.30	^79^
Cellulose-based biocomposite film	146.81	^80^
Activated spent tea (AST)	104.20	^81^
Sludge-rice husk biochar	22.59	^82^
Mandarin nanoporous carbon	313.00	^83^
Jute stick biomass	198.86	^84^
Beer waste-activated carbon	341.00	^85^
Pomegranate peel	364.75	^86^
Carbonized mandarin peel	196.08	^87^
Jute stick-derived activated carbon	384.60	^88^
Banana stem-activated carbon	64.66	^89^
Coconut leaf	357.14	^90^
Macore fruit shells	10.61	^91^
SDBA	643.74	This work

### Regeneration of SDBA

**Figure 15:**
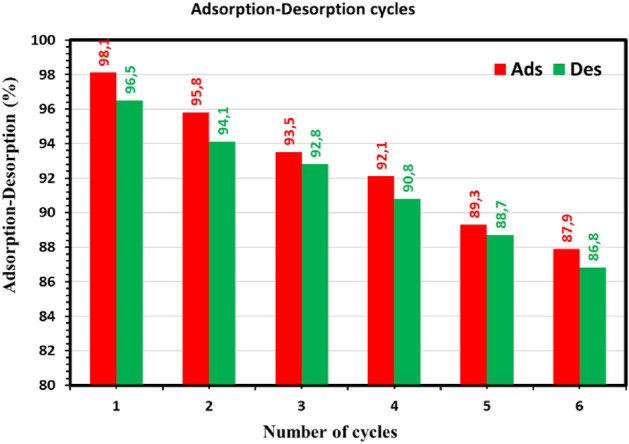
MB dye was desorption% from SDBA by 0.1 M HCl and NaOH, and SDBA regeneration was used to control MB dye removal cycles (100 mg/L MB dye, 1.0 g/L SDBA).

The outcomes of the regeneration experiment are covered in this section. Due to the expense of adsorption, recycling SDBA is very necessary. Adsorbent dosage increases the expense of the water treatment process in dye removal treatment plants^92,93^. Hence, reusability offers value by lowering operational costs, namely the cost of the adsorbent as an input, for adsorbents like activated carbon that entail production expenses. It is also a desirable step for the adsorbent to regenerate easily. In order to test the SDBA's reusability for MB adsorption across six cycles, 0.1 M NaOH and 0.1 N HCl solutions were used. The six adsorption–desorption cycles of the MB adsorption capacity using SDBA are shown in Fig. 15. Figure 15 shows that SDBA's adsorption capacity decreased after each regeneration cycle. The percentage of MB removal (Ads%) was 98.1% during the first regeneration cycle. However, after six cycles, the efficiency decreased to 87.9%, indicating that the regenerated adsorbent still has a high potential for MB removal and is appropriate for repeated use.

## Conclusion

This study aimed to develop a new adsorbent (sawdust biochar, SDBA) for removing Methylene Blue (MB) dye from an aqueous solution. Sawdust, an agricultural waste material, was modified with ammonia and used as an effective bio-sorbent. According to the textural characterisation, the biochars are largely microporous, and SDBA has a BET surface area of 2.88 m^2^/g. By using SEM examination, the well-developed porosity structure was confirmed. The results of this investigation demonstrated the importance of the pH of the solution, the starting concentration of MB dye, the mass of the adsorbent, and the adsorption period in the process of adsorbing MB dye onto manufactured SDBA adsorbent. The MB dye removal % rose with pH up to pH 8, and contact time and initial MB dye concentration rise also had a favorable impact on the removal. The maximum elimination of MB dye and the least amount of adsorption (*q*_*e*_) at equilibrium were determined by working with an SDBA mass of 1.0 g/L concentration. The equilibrium data were best described by the Freundlich (FIM) and Langmuir (FIM) isotherm models. LIM and FIM in error functions comparison determined the most suitable isotherm models. The Freundlich constant, *n*_*F*_, indicates that the adsorption was favorable and physical. According to FIM, the maximum adsorption capacity (Qm) was 643.74 mg/g. The kinetics of the process is best explained using a pseudo-second-order (PSOM) kinetics model with higher *R*^2^ (Table 5), which suggests that the process was controlled by chemisorption. This study demonstrates that SDBA is a promising bio-sorbent for eliminating MB dye from wastewater.

## Data Availability

The datasets used in this investigation are accessible for review upon request from the paper's corresponding author.

## References

[1] Adeniyi, A. G. & Ighalo, J. O. Biosorption of pollutants by plant leaves: An empirical review (2019).

[2] Liang L, Zhang S, Goenaga GA, Meng X, Zawodzinski TA, Ragauskas AJ (2020). Chemically cross-linked cellulose nanocrystal aerogels for effective removal of cation dye. Front Chem..

[3] Yu L, Gamliel DP, Markunas B, Valla JA (2021). A promising solution for food waste: Preparing activated carbons for phenol removal from water streams. ACS Omega.

[4] Lin D, Wu F, Hu Y, Zhang T, Liu C, Hu Q, Hu Y, Xue Z, Han H, Ko TH (2020). Adsorption of dye by waste black tea powder: Parameters, kinetic, equilibrium, and thermodynamic studies. J. Chem..

[5] Al-Mamun MR, Karim MN, Nitun NA, Kader S, Islam MS, Khan MZH (2021). Photocatalytic performance assessment of GO and Ag co-synthesized TiO_2_ nanocomposite for the removal of methyl orange dye under solar irradiation. Environ. Technol. Innov..

[6] You X, Wang R, Zhu Y, Sui W, Cheng D (2021). Comparison of adsorption properties of a cellulose-rich modified rice husk for the removal of methylene blue and aluminum (III) from their aqueous solution. Ind. Crops. Prod..

[7] Li Q, Zhao Y, Wang L, Aiqin W (2011). Adsorption characteristics of methylene blue onto the N-succinyl-chitosan-g-polyacrylamide/attapulgite composite. Korean J. Chem. Eng..

[8] Sabri AA, Albayati TM, Alazawi RA (2015). Synthesis of ordered mesoporous SBA-15 and its adsorption of methylene blue. Korean J. Chem. Eng..

[9] Bhakta AK, Kumari S, Hussain S, Detriche S, Delhalle J, Mekhalif Z (2019). Differently substituted aniline functionalized MWCNTs to anchor oxides of Bi and Ni nanoparticles. J. Nanostructure Chem..

[10] Corda NC, Kini MS (2018). A review on adsorption of cationic dyes using activated carbon. MATEC Web Conf..

[11] Holkar, C. R., Jadhav, A. J., Pinjari, D. V., Mahamuni, N. M. & Pandit, A. B. A critical review on textile wastewater treatments: Possible approaches (2016) 10.1016/j.jenvman.2016.07.090 27497312

[12] Ahmad AA, Al-Raggad M, Shareef N (2021). Production of activated carbon derived from agricultural by-products via microwave-induced chemical activation: A review. Carbon Lett..

[13] Pallarés J, González-Cencerrado A, Arauzo I (2018). Production and characterization of activated carbon from barley straw by physical activation with carbon dioxide and steam. Biomass Bioenergy.

[14] Zhu J, Li Y, Xu L, Liu Z (2018). Removal of toluene from waste gas by adsorption-desorption process using corncob-based activated carbons as adsorbents. Ecotoxicol. Environ. Saf..

[15] Dhahri R, Yılmaz M, Mechi L, Alsukaibi AKD, Alimi F, Ben Salem R, Moussaoui Y (2022). Optimization of the preparation of activated carbon from prickly pear seed cake for the removal of lead and cadmium ıons from aqueous solution. Sustainability..

[16] Georgin J, da Boit Martinello K, Franco DSP, Netto MS, Piccilli DGA, Yilmaz M, Silva LFO, Dotto GL (2022). Residual peel of pitaya fruit (*Hylocereus undatus*) as a precursor to obtaining an efficient carbon-based adsorbent for the removal of metanil yellow dye from water. J. Environ. Chem. Eng..

[17] Jabar JM, Adebayo MA, Owokotomo IA, Odusote YA, Yılmaz M (2022). Synthesis of high surface area mesoporous ZnCl_2_–activated cocoa (*Theobroma cacao* L.) leaves biochar derived via pyrolysis for crystal violet dye removal. Heliyon.

[18] Yang Y, Phuong Nguyen TM, Van HT, Nguyen QT, Nguyen TH, Lien Nguyen TB, Hoang LP, Van Thanh D, Nguyen TV, Nguyen VQ, Thang PQ, Yılmaz M, Le VG (2022). ZnO nanoparticles loaded rice husk biochar as an effective adsorbent for removing reactive red 24 from aqueous solution. Mater. Sci. Semicond. Process.

[19] Yılmaz M, Eldeeb TM, Hassaan MA, El-Nemr MA, Ragab S, El Nemr A (2022). The use of mandarin-biochar-O3-TETA (MBT) produced from mandarin peels as a natural adsorbent for the removal of acid red 35 (AR35) dye from water. Environ. Processes..

[20] Tsoncheva T, Mileva A, Tsyntsarski B, Paneva D, Spassova I, Kovacheva D, Velinov N, Karashanova D, Georgieva B, Petrov N (2018). Activated carbon from Bulgarian peach stones as a support of catalysts for methanol decomposition. Biomass Bioenergy..

[21] Ahmed MB, Hasan Johir MA, Zhou JL, Ngo HH, Nghiem LD, Richardson C, Moni MA, Bryant MR (2019). Activated carbon preparation from biomass feedstock: Clean production and carbon dioxide adsorption. J. Clean. Prod..

[22] Ait Ahsaine H, Zbair M, Anfar Z, Naciri Y, El Haouti R, El Alem N, Ezahri M (2018). Cationic dyes adsorption onto high surface area ‘almond shell’ activated carbon: Kinetics, equilibrium isotherms and surface statistical modeling. Mater. Today Chem..

[23] Corral-Bobadilla M, Lostado-Lorza R, Somovilla-Gómez F, Escribano-García R (2021). Effective use of activated carbon from olive stone waste in the biosorption removal of Fe(III) ions from aqueous solutions. J. Clean. Prod..

[24] Abatan OG, Oni BA, Agboola O, Efevbokhan V, Abiodun OO (2019). Production of activated carbon from African star apple seed husks, oil seed and whole seed for wastewater treatment. J. Clean. Prod..

[25] El-Nemr MA, Yılmaz M, Ragab S, El Nemr A (2022). Biochar-SO prepared from pea peels by dehydration with sulfuric acid improves the adsorption of Cr^6+^ from water. Biomass Convers. Biorefin..

[26] El-Nemr MA, Aigbe UO, Hassaan MA, Ukhurebor KE, Ragab S, Onyancha RB, Osibote OA, El Nemr A (2022). The use of biochar-NH_2_ produced from watermelon peels as a natural adsorbent for the removal of Cu(II) ion from water. Biomass Convers. Biorefin..

[27] Wang L, Wang Y, Ma F, Tankpa V, Bai S, Guo X, Wang X (2019). Mechanisms and reutilization of modified biochar used for removal of heavy metals from wastewater: A review. Sci. Total Environ..

[28] Chang Z, Tian L, Wu M, Dong X, Peng J, Pan B (2018). Molecular markers of benzene polycarboxylic acids in describing biochar physiochemical properties and sorption characteristics. Environ. Pollut..

[29] Hassaan MA, Nemr AE, Elkatory MR, Eleryan A, Ragab S, Sikaily AE, Pantaleo A (2021). Enhancement of biogas production from macroalgae *Ulva **latuca* via ozonation pretreatment. Energies.

[30] Zhang H, Hay AG (2020). Magnetic biochar derived from biosolids via hydrothermal carbonization: Enzyme immobilization, immobilized-enzyme kinetics, environmental toxicity. J. Hazard. Mater..

[31] Ma Y, Liu WJ, Zhang N, Li YS, Jiang H, Sheng GP (2014). Polyethylenimine modified biochar adsorbent for hexavalent chromium removal from the aqueous solution. Bioresour. Technol..

[32] Chen Z, Xiao X, Chen B, Zhu L (2014). Quantification of chemical states, dissociation constants, and contents of oxygen-containing groups on the surface of biochars produced at different temperatures. Environ. Sci. Technol..

[33] Fuertes AB, Arbestain MC, Sevilla M, Macia-Agullo JA, Fiol S, Lopez R, Smernik RJ, Aitkenhead WP, Arce F, Macias F (2010). Chemical and structural properties of carbonaceous products obtained by pyrolysis and hydrothermal carbonization of corn stover. Soil. Res..

[34] Wang S, Gao B, Zimmerman AR, Li Y, Ma L, Harris WG, Migliaccio KW (2015). Removal of arsenic by magnetic biochar prepared from pinewood and natural hematite. Bioresour. Technol..

[35] Sun K, Kang M, Zhang Z, Jin J, Wang Z, Pan Z, Xu D, Wu F, Xing B (2013). Impact of deashing treatment on biochar structural properties and potential sorption mechanisms of phenanthrene. Environ. Sci. Technol..

[36] Wang P, Liu X, Wu X, Xu J, Dong F, Zheng Y (2018). Evaluation of biochars in reducing the bioavailability of flubendiamide in water/sediment using passive sampling with polyoxymethylene. J. Hazard. Mater..

[37] Xu RK, Xiao SC, Yuan JH, Zhao AZ (2011). Adsorption of methyl violet from aqueous solutions by the biochars derived from crop residues. Bioresour. Technol..

[38] Hassaan MA, Yilmaz M, El Nemr A (2023). Advanced oxidation process for treatment of water containing mixture of Acid Red 17, Acid Yellow 11, Direct Yellow 12, Direct Blue 86 and Mordant Violet 40 dyes. AKÜ FEMÜBİD.

[39] Gregg SJ, Sing KSW (1982). Adsorption Surface Area and Porosity.

[40] Rouquerol F, Rouquerol J, Sing KSW (1999). Adsorption by Powders and Porous Solids.

[41] Barrett EP, Joyner LG, Halenda PP (1951). The determination of pore volume and area distributions in porous substances. I. Computations from nitrogen isotherms. J. Am. Chem. Soc..

[42] El Nemr MA, Yılmaz M, Ragab S, Hassaan MA, El Nemr A (2023). Isotherm and kinetic studies of acid yellow 11 dye adsorption from wastewater using *Pisum sativum* peels microporous activated carbon. Sci. Rep..

[43] Eleryan A, Hassaan MA, Aigbe UO, Ukhurebor KE, Onyancha RB, El-Nemr MA, Ragab S, Hossain I, El Nemr A (2023). Kinetic and isotherm studies of Acid orange 7 dye absorption using sulfonated mandarin biochar treated with TETA. Biomass Convers. Biorefin..

[44] Eleryan A, Aigbe UO, Ukhurebor KE, Onyancha RB, Hassaan MA, Elkatory MR, Ragab S, Osibote OA, Kusuma HS, El Nemr A (2023). Direct blue 106 dye adsorption using green synthesized zinc oxide nanoparticles. Environ. Sci. Pollut. Res..

[45] Eleryan A, Aigbe UO, Ukhurebor KE, Onyancha RB, Eldeeb TM, El-Nemr MA, Hassaan MA, Ragab S, Osibote OA, Kusuma HS, Darmokoesoemo H, El Nemr A (2022). Copper (II) ion removal by chemically and physically modified sawdust biochar. Biomass Convers. Biorefin..

[46] Eldeeb TM, Aigbe UO, Ukhurebor KE, Onyancha RB, El-Nemr MA, Hassaan MA, Ragab S, Osibote OA, El Nemr A (2022). Adsorption of methylene blue dye on sawdust ozone, purified sawdust, and sonicated sawdust biochars. Biomass Convers. Biorefin..

[47] El Nemr A, Shoaib AG, El Sikaily A, Mohamed AEDA, Hassan AF (2021). Evaluation of cationic methylene blue dye removal by high surface area mesoporous activated carbon derived from *Ulva lactuca*. Environ. Process..

[48] El Nemr A, El-Sikaily A, Khaled A (2010). Modeling of adsorption isotherms of Methylene Blue onto rice husk activated carbon. Egypt. J. Aquat. Res..

[49] El Nemr A, Aboughaly RM, El Sikaily A, Masoud MS, Ramadan MS, Ragab S (2022). Microporous activated carbons with a high surface area of type I adsorption isotherm derived from sugarcane bagasse impregnated with zinc chloride. Carbon Lett..

[50] Chakraborty TK, Islam MS, Zaman S, Kabir AHME, Ghosh GC (2020). Jute (*Corchorus olitorius*) stick charcoal as a low-cost adsorbent for the removal of methylene blue dye from aqueous solution. SN Appl. Sci..

[51] El-Nemr MA, Abdelmonem NM, Ismail IMA, Ragab S, El Nemr A (2020). The efficient removal of the hazardous Azo Dye Acid Orange 7 from water using modified biochar from Pea peels. Desalin. Water Treat..

[52] El-Nemr MA, Abdelmonem NM, Ismail IMA, Ragab S, El Nemr A (2020). Removal of Acid Yellow 11 Dye using novel modified biochar derived from Watermelon Peels. Desalin. Water Treat..

[53] El-Nemr MA, Ismail IMA, Abdelmonem NM, El Nemr A, Ragab S (2021). Amination of biochar derived from watermelon peel by triethylenetetramine and ammonium hydroxide for toxic chromium removal enhancement. Chin. J. Chem. Eng..

[54] Eleryan A, Aigbe UO, Ukhurebor KE, Onyancha RB, Eldeeb TM, El-Nemr MA, Hassaan MA, Ragab S, Osibote OA, Kusuma HS, Darmokoesoemo H, El Nemr A (2022). Copper (II) ion removal by chemically and physically modified sawdust biochar. Biomass Convers. Biorefin..

[55] Hassaan MA, El Nemr A, Elkatory MR, Ragab S, El-Nemr MA, Pantaleo A (2021). Synthesis, characterization, and synergistic effects of modified biochar in combination with α-Fe_2_O_3_ NPs on biogas production from red algae *Pterocladia*
*capillacea*. Sustainability.

[56] Crini G, Peindy HN, Gimbert F, Robert C (2007). Removal of C.I. Basic Green 4 (Malachite Green) from aqueous solutions by adsorption using cyclodextrin-based adsorbent: Kinetic and equilibrium studies. Sep. Purif. Technol..

[57] Kim M, Choong CE, Hyun S, Park CM, Lee G (2020). Mechanism of simultaneous removal of aluminum and fluoride from aqueous solution by La/Mg/Si-activated carbon. Chemosphere.

[58] Guiza S, Bagane M, Al-Soudani AH, Amore H (2016). Ben: Adsorption of basic dyes onto natural clay. Desalination.

[59] El-Nemr MA, Yılmaz M, Ragab S, Hassaan MA, El Nemr A (2023). Isotherm and kinetic studies of acid yellow 11 dye adsorption from wastewater using *Pisum sativum* peels microporous activated carbon. Sci. Rep..

[60] Hameed BH, Tan IAW, Ahmad AL (2008). Adsorption isotherm, kinetic modeling and mechanism of 2,4,6-trichlorophenol on coconut husk-based activated carbon. Chem. Eng. J..

[61] Langmuir I (1918). The adsorption of gases on plane surfaces of glass, mica and platinum. J. Am. Chem. Soc..

[62] Freundlich HMF (1906). Over the adsorption in solution. J. Phys. Chem..

[63] Tempkin MI, Pyzhev V (1940). Kinetics of ammonia synthesis on promoted iron catalyst. Acta Phys. Chim. USSR.

[64] Vijayaraghavan K, Padmesh TVN, Palanivelu K, Velan M (2006). Biosorption of nickel(II) ions onto *Sargassum wightii*: Application of two-parameter and three-parameter isotherm models. J. Hazard Mater..

[65] Senthil Kumar P, Ramalingam S, Senthamarai C, Niranjanaa M, Vijayalakshmi P, Sivanesan S (2010). Adsorption of dye from aqueous solution by cashew nut shell: Studies on equilibrium isotherm, kinetics and thermodynamics of interactions. Desalination.

[66] Fytianos K, Voudrias E, Kokkalis E (2000). Sorption-desorption behaviour of 2,4-dichlorophenol by marine sediments. Chemosphere.

[67] Ambaye TG, Vaccari M, van Hullebusch ED, Amrane A, Rtimi S (2021). Mechanisms and adsorption capacities of biochar for the removal of organic and inorganic pollutants from industrial wastewater. Int. J. Environ. Sci. Technol..

[68] Ahmad M, Rajapaksha AU, Lim JE, Zhang M, Bolan N, Mohan D, Vithanage M, Lee SS, Ok YS (2014). Biochar as a sorbent for contaminant management in soil and water: A review. Chemosphere.

[69] Zheng H, Wang Z, Zhao J (2013). Sorption of antibiotic sulfamethoxazole varies with biochars produced at different temperatures. Environ. Pollut..

[70] Mukherjee A, Zimmerman AR, Harris W (2011). Surface chemistry variations among a series of laboratory-produced biochars. Geoderma.

[71] Inyang M, Gao B, Zimmerman A, Zhang M, Chen H (2014). Synthesis, characterization, and dye sorption ability of carbon nanotube–biochar nanocomposites. Chem. Eng. J..

[72] El Nemr A, El-Sikaily A, Khaled A, Abdelwahab O (2015). Removal of toxic chromium from aqueous solution, wastewater and saline water by marine red alga *Pterocladia capillacea* and its activated carbon. Arab. J. Chem..

[73] Li M, Liu Q, Guo L, Zhang Y, Lou Z, Wang Y, Qian G (2013). Cu(II) removal from aqueous solution by *Spartina alterniflora* derived biochar. Bioresour. Technol..

[74] El-Khaiary MI, Malash GF (2011). Common data analysis errors in batch adsorption studies. Hydrometallurgy.

[75] Hameed BH, Daud FBM (2008). Adsorption studies of basic dye on activated carbon derived from agricultural waste: *Hevea brasiliensis* seed coat. Chem. Eng. J..

[76] Weber WJ, Morris JC (1963). Kinetics of adsorption on carbon from solution. J. Sanitary Eng. Division.

[77] Parshetti GK, Hoekman SK, Balasubramanian R (2013). Chemical, structural and combustion characteristics of carbonaceous products obtained by hydrothermal carbonization of palm empty fruit bunches. Bioresour. Technol..

[78] Tsai WT, Chen HR (2013). Adsorption kinetics of herbicide paraquat in aqueous solution onto a low-cost adsorbent, swine-manure-derived biochar. Int. J. Environ. Sci. Technol..

[79] Ahmed MJ, Theydan SK (2014). Optimization of microwave preparation conditions for activated carbon from *Albizia lebbeck* seed pods for methylene blue dye adsorption. J. Anal. Appl. Pyrolysis..

[80] Somsesta N, Piyamawadee C, Sricharoenchaikul V, Aht-Ong D (2020). Adsorption isotherms and kinetics for the removal of cationic dye by cellulose-based adsorbent biocomposite films. Korean J. Chem. Eng..

[81] Babaei AA, Khataee A, Ahmadpour E, Sheydaei M, Kakavandi B, Alaee Z (2016). Optimization of cationic dye adsorption on activated spent tea: Equilibrium, kinetics, thermodynamic and artificial neural network modeling. Korean J. Chem. Eng..

[82] Chen S, Qin C, Wang T, Chen F, Li X, Hou H, Zhou M (2019). Study on the adsorption of dyestuffs with different properties by sludge-rice husk biochar: Adsorption capacity, isotherm, kinetic, thermodynamics and mechanism. J. Mol. Liq..

[83] Koyuncu F, Güzel F (2021). Use of new nanoporous carbon produced from Mandarin (*Citrus reticulata*) industrial processing waste to remove anionic and cationic dyes. Sep. Sci. Technol..

[84] Ghosh RK, Ray DP, Debnath S, Tewari A, Das I (2019). Optimization of process parameters for methylene blue removal by jute stick using response surface methodology. Environ. Prog. Sustain Energy..

[85] Hao W, Björkman E, Lilliestråle M, Hedin N (2014). Activated carbons for water treatment prepared by phosphoric acid activation of hydrothermally treated beer waste. Ind. Eng. Chem. Res..

[86] Jawad AH, Sauodi MH, Mastuli MS, Aouda MA, Radzun KA (2018). Pomegranate peels collected from fresh juice shop as a renewable precursor for high surface area activated carbon with potential application for methylene blue adsorption. Desalin. Water Treat..

[87] Unugul T, Nigiz FU (2020). Preparation and characterization an active carbon adsorbent from waste mandarin peel and determination of adsorption behavior on removal of synthetic dye solutions. Water Air Soil Pollut..

[88] Ghosh RK, Ray DP, Tewari A, Das I (2021). Removal of textile dyes from water by jute stick activated carbon: Process optimization and isotherm studies. Int. J. Environ. Sci. Technol..

[89] Danish M, Ahmad T, Nadhari WNAW, Ahmad M, Khanday WA, Ziyang L, Pin Z (2018). Optimization of banana trunk-activated carbon production for methylene blue-contaminated water treatment. Appl. Water Sci..

[90] Pourmortazavi SM, Rahimi-Nasrabadi M, Aghazadeh M, Ganjali MR, Karimi MS, Norouzi P (2017). Optimized routes for the preparation of gadolinium carbonate and oxide nano-particles and exploring their photocatalytic activity. Desalin. Water Treat..

[91] Aboua KN, Yobouet YA, Yao KB, Goné DL, Trokourey A (2015). Investigation of dye adsorption onto activated carbon from the shells of Macoré fruit. J. Environ. Manag..

[92] Bhomick PC, Supong A, Baruah M, Pongener C, Gogoi C, Sinha D (2020). Alizarin Red S adsorption onto biomass-based activated carbon: Optimization of adsorption process parameters using Taguchi experimental design. Int. J. Environ. Sci. Technol..

[93] Hannachi Y, Hafidh A (2020). Biosorption potential of *Sargassum muticum* algal biomass for methylene blue and lead removal from aqueous medium. Int. J. Environ. Sci. Technol..

